# Lab-on-Valve Automated and Miniaturized Assessment of Nanoparticle Concentration Based on Light-Scattering

**DOI:** 10.1021/acs.analchem.2c04631

**Published:** 2023-02-21

**Authors:** Sara S. Marques, Inês I. Ramos, Carla Silva, Luisa Barreiros, Maria R. Domingues, Marcela A. Segundo

**Affiliations:** † LAQV, REQUIMTE, University of Porto, Department of Chemical Sciences, Faculty of Pharmacy, R. Jorge Viterbo Ferreira 228, 4050-313 Porto, Portugal; ‡ Centre of Biological Engineering (CEB), University of Minho, 4710-057 Braga, Portugal; # LABBELS - Associate Laboratory, 4710-057 Braga, Guimarães Portugal; § School of Health, Polytechnic Institute of Porto, R. Dr. António Bernardino de Almeida 400, 4200-072 Porto, Portugal; ∥ CESAM-Centre for Environmental and Marine Studies, Department of Chemistry, Santiago University Campus, University of Aveiro, 3810-193 Aveiro, Portugal; ⊥ Mass Spectrometry Centre, LAQV REQUIMTE, Department of Chemistry, Santiago University Campus, University of Aveiro, 3810-193 Aveiro, Portugal

## Abstract

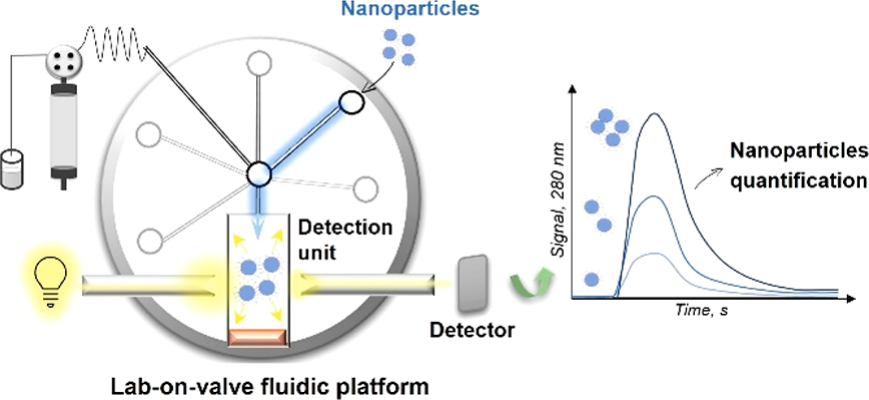

Nanoparticles (NPs) concentration directly impacts the dose delivered to target tissues by nanocarriers. The evaluation of this parameter is required during NPs developmental and quality control stages, for setting dose–response correlations and for evaluating the reproducibility of the manufacturing process. Still, faster and simpler procedures, dismissing skilled operators and post-analysis conversions are needed to quantify NPs for research and quality control operations, and to support result validation. Herein, a miniaturized automated ensemble method to measure NPs concentration was established under the lab-on-valve (LOV) mesofluidic platform. Automatic NPs sampling and delivery to the LOV detection unit were set by flow programming. NPs concentration measurements were based on the decrease in the light transmitted to the detector due to the light scattered by NPs when passing through the optical path. Each analysis was accomplished in 2 min, rendering a determination throughput of 30 h^–1^ (6 samples h^–1^ for *n* = 5) and only requiring 30 μL (≈0.03 g) of NPs suspension. Measurements were performed on polymeric NPs, as these represent one of the major classes of NPs under development for drug-delivery aims. Determinations for polystyrene NPs (of 100, 200, and 500 nm) and for NPs made of PEGylated poly-d,l-lactide-*co*-glycolide (PEG–PLGA, a biocompatible FDA-approved polymer) were accomplished within 10^8^–10^12^ particles mL^–1^ range, depending on the NPs size and composition. NPs size and concentration were maintained during analysis, as verified for NPs eluted from the LOV by particle tracking analysis (PTA). Moreover, concentration measurements for PEG–PLGA NPs loaded with an anti-inflammatory drug, methotrexate (MTX), after their incubation in simulated gastric and intestinal fluids were successfully achieved (recovery values of 102–115%, as confirmed by PTA), showing the suitability of the proposed method to support the development of polymeric NPs targeting intestinal delivery.

The advent of drug nanocarriers provided a means to modulate the solubility, stability, and delivery profile of pharmaceutical drugs.^1,2^ Such strategies afforded new solutions for improving the therapeutic index of diverse drug molecules, often limited by bioavailability and/or toxicity issues, expanding the therapeutic opportunities for several health conditions.

These promises gave rise to the extensive development of different and increasingly complex nanoproducts over the last 25 years. Still, the regulatory approval of drug delivery nanoparticles (NPs) requires a thorough and robust characterization of their main physicochemical properties, along with evidence of reproducible manufacture.^3,4^

NPs concentration (defined as the number of NPs per volume of formulation)^5^ and drug encapsulation efficiency (quantity of drug associated with the NPs in relation to the total amount of drug)^6^ are among the critical parameters to determine the dose delivered to target tissues, and consequently, for the therapeutic response.^7^ Accurate assessment of NPs concentration is required to set dose–response correlations during formulation design and is a requisite at industrial level to control the reproducibility of NPs manufacturing batches and to comply with current^3,8^ and future needs at quality control and regulatory levels.^9^

Still, measuring NPs concentration resorting to the currently available techniques poses several challenges,^5,9^ namely, for organic NPs, whose mass cannot be quantified by inductively coupled plasma mass spectrometry,^10^ as occurs for inorganic NPs.^10,11^ Therefore, for organic NPs, concentration measurements are mainly performed by single-particle analysis techniques, such as particle tracking analysis (PTA) and tunable resistive pulse sensing (TRPS).^9,12^ PTA measurements result from light-scattering-based tracking of individual NPs in a given illumination volume. However, PTA analysis requires extensive sample dilution, which can induce NPs aggregation, causing inaccurate measurements.^5,13^ Also, results depend on the parameters set for video acquisition and processing, which are operator-dependent, thus requiring trained personnel and often suffering from variability if undertaken by different operators.^9,13^ TRPS is a more robust technique, in which NPs concentration is assessed while they pass through a porous membrane, driven by voltage application.^9,12^ Nevertheless, TRPS requires NPs dilution in electrolytes, analysis at different pressure points, calibration with particle standards prior to each sample analysis and for each pressure point, and experienced operators^9,12^ capable of optimizing measurement parameters, which renders the technique not straightforward for routine applications at industrial or medical facilities. Considering these limitations, other techniques, such as nano flow cytometry (nFC), multiangle light scattering (MALS), and differential centrifugal sedimentation (DCS), have been also used for measuring NPs concentration, and/or to provide a means for results validation. In nFC, single-particle counting is performed by light scattering (label free) and/or fluorescence measurements after NPs irradiation by a laser beam.^14^ Still, light-scattering measurements for NPs with <300 nm are challenging and were only performed so far resorting to custom-made, not broadly available nFC setups,^14,15^ while fluorescence detection requires NPs labeling and purification before analysis. Regarding concentration measurements by ensemble techniques, such as MALS and DCS, both require post-analysis conversions of the obtained NPs size distributions, which entails NPs properties, such as size, density, refractive index, and media viscosity to be well-known for accurate estimations.^9,16^

Considering the above-mentioned challenges, improved, alternative, and orthogonal procedures to the currently available strategies for NPs concentration measurements have been pursued.^17,18^ Nevertheless, label free, simpler, and fit for purpose methods suitable to characterize the number of organic NPs in formulation batches for routine quality control operations are yet required. In this context, an automated, miniaturized and simple method to quantify NPs resorting to the sequential injection lab-on-valve (SI-LOV) system^19,20^ is proposed. LOV is a mesofluidic platform that provides precise handling of small volumes (μL) of samples/reagents in rigid miniaturized channels resorting to flow programming and to a multiposition selection valve,^20,21^ thus affording robust and reproducible operations with downscaled consumption of samples/reagents.^21^ LOV accommodates sampling, online dilution and mixing by flow reversal, and online detection as it integrates an optical detection unit, enabling several operations in a single setup. This system has successfully been employed to set simpler, faster and less-laborious quantification of small molecules,^22^ sorbent-based sample pretreatment,^23,24^ and molecular recognition procedures.^19,25^ Furthermore, considering the need of ensuring minimal stress and dilution during the analysis of organic NPs, the LOV platform emerges as a valuable alternative for NPs characterization as it offers (i) inert bore conduits (*ca*. 1.0 mm), larger than conventional size-exclusion columns and ultrafiltration/dialysis membranes, (ii) low operation flow rates (*e.g*., 1 μL s^–1^), and (iii) short distance (10 mm) between the sampling port and the detection unit.

Given the previous context, in this work, a miniaturized and nondestructive methodology was set under the LOV to evaluate the concentration of polystyrene NPs with different diameters (100, 200, and 500 nm). The developed framework was used to determine the concentration of empty (i.e., unloaded) and methotrexate (MTX)-loaded PEGylated poly-d,l-lactide-*co*-glycolide (PEG–PLGA) NPs as PEG–PLGA NPs are among the most produced due to the biocompatibility, biodegradability, and “stealth” properties conferred by the PEG–PLGA polymer.^26^ Moreover, the application of the established method was further extended to assess NPs stability when exposed to surrogate biological media, to investigate NPs suitability for oral intake.

## Experimental Section

### Reagents and Solutions

Details on the reagents and salts used for preparing the PEG–PLGA NPs, the phosphate-buffered saline solution (PBS) used as carrier in the SI-LOV system and for diluting NPs suspensions, and the simulated gastric and intestinal fluids are provided in Supporting Information, along with preparation details.

### Polymeric Nanoparticles

Polystyrene NP standards with monodisperse distributions, with 188 ± 4, 102 ± 3, and 502 ± 13 nm of diameter, and with estimated particle concentrations^7^ of 5.5, 34, and 0.29 × 10^12^ particles mL^–1^ were acquired from Sigma-Aldrich (St. Louis, MO, USA). These were designated as NPs A, B, and C for simplicity. Different concentration levels were prepared from each standard by adequate gravimetric dilution in PBS buffer.

PEG–PLGA NPs were prepared weekly by the single emulsion-solvent evaporation technique^27^ (details on Supporting Information). Their hydrodynamic diameter and polydispersity index (PDI) were characterized by dynamic light scattering (DLS, ZetaPALS Particle Analyzer, Brookhaven Instrument Corps, Santa Barbara, CA) after preparation and after NPs purification (details on Supporting Information). Moreover, the size and concentration of PEG–PLGA NPs final suspension were characterized resorting to particle tracking analysis (PTA, NS500, Malvern Panalytical, Malvern, UK). For this, NPs were diluted 16200× in ultrapure water prior to analysis. A 60 s video (*n* ≥ 3) was recorded for the suspensions of empty and loaded NPs (data will be available upon request). Videos were processed using the NTA 2.3 software (Malvern Panalytical).

### Nanoparticles Quantification by Sequential Injection on the Lab-on-Valve (SI-LOV)

The configuration of the SI-LOV (MicroSIA, FIAlab instruments, Inc., Bellevue, WA, USA) system used for nanoparticles quantification (Figure S1) is described in Supporting Information. Optical detection and quantification of nanoparticles was performed in the LOV integrated detection unit (further details on Supporting Information)^19,25,28^ by measuring the light attenuation (i.e., light lost due to scattering and/or absorbance events) when nanoparticles were present in the optical path.

The analytical routine comprised five steps (Table S1): first, the syringe pump was filled with carrier solution (PBS). Subsequently, 20 μL of carrier was dispensed into the flow cell for performing a reference scan. Then, the multiposition valve was switched to the sample port, and the sample was aspirated into the holding coil at 3 μL s^–1^. After flow reversal, the NPs present in the holding coil were directed at 2 μL s^–1^ to the detection unit, where the light attenuation values were monitored at 280, 302, 320, and 480 nm. These last two steps can be repeated (*e.g*., up to five times) for replicate analysis of the same sample. Finally, the carrier remaining in the syringe pump was discarded to waste to clean the holding coil and to prepare the system for the measurement of the following sample.

Different concentrations of polystyrene NPs (A, B, and C) and of PEG–PLGA and PEG–PLGA–MTX NPs were analyzed following this analytical routine. For this, working solutions were prepared by submitting the NPs stock suspensions to vortex (3000 rpm) for 30 s, followed by adequate dilution in PBS. Each working solution was submitted to vortex immediately before its introduction into the LOV channel, which was just performed prior the start of each analytical cycle. Likewise, PEG–PLGA-MTX NPs were also analyzed after incubation for 2 h with simulated gastric fluid (containing or not pepsin), and for 4 h with simulated intestinal fluid. Further experimental details on analysis conditions, control experiments and on the data analysis performed for calculating peak height, peak area, peak width, and method analytical features (*e.g*., linearity, limits of detection (LOD) and quantification (LOQ), precision, and accuracy)^29^ are provided at Supporting Information.

## Results and Discussion

### Nanoparticle Quantification Based on Light Scattering under Dynamic Flow Injection

NPs concentration measurements were based on the decrease of light reaching the detector when part of the incident light was scattered by NPs passing through the LOV flow cell, for negligible absorbance events^30^ (*e.g*., PEG–PLGA materials). A brief overview on the fundamentals of NPs light scattering can be found in Supporting Information. Herein, as incident wavelengths (λ_inc_) from 280–500 nm and NPs with 100–500 nm of diameter were targeted, scattering events were putatively considered to obeyed to Mie theory.^30,31^ Thus, the total light scattered is a function of (i) NPs size, (ii) NPs concentration, (iii) NPs extinction efficiency (*Q*_ext_), and (iv) the optical path as described by equation S1, with *Q*_ext_ being dependent on the λ_inc_, and on the differences between NPs’ material and surrounding medium refractive indexes.^30,31^

In this work, although data acquisition was performed at 4 wavelengths, light attenuation measurements were taken at 280 nm, as this wavelength provided increased sensitivity in relation to higher λ_inc_ for all the NPs under study (Figure S2) since the magnitude of scattering intensity is inversely proportional to λ_inc_.^31^

Moreover, the measurements were performed under sequential injection (SI) conditions, as these offer reproducible features regarding the maintenance of NPs in suspension as they pass through the detector, beyond miniaturization. Since several variables will affect the dispersion of NPs in the carrier fluid, namely, the flow rate^32^ and the sample volume,^33^ these parameters were further studied to implement a reliable analytical routine for NPs quantification.

#### Effect of Flow Rate

Figure S3 depicts the signal profiles (light attenuation vs. time) when a fixed volume (30 μL) of polystyrene NPs A (188 ± 4 nm) was aspirated from the sampling port and sent to the detection unit at different flow rates (1, 2, and 4 μL s^–1^). Good repeatability concerning signal height (RSD < 2%) and area (RSD < 5%) was observed independently of the flow rate tested. Nevertheless, experiments at the lowest flow rate (1 μL s^–1^) resulted in the broadest peak profile and in the lowest signal height (0.304 ± 0.004), which was caused by the increased NPs dispersion in the carrier when the time of sample displacement from the holding coil to the detector increases.^32^ Experiments at 4 μL s^–1^ resulted in the narrowest peak, with a peak height (0.35 ± 0.1) similar to the obtained for experiments at 2 μL s^–1^ (0.355 ± 0.002), although with RSD values at least twice larger for peak height and area. This fact is likely attributed to the faster passage of NPs through the detector, causing some irreproducibility probably due to inadequacy in data frequency acquisition.^34^ Thus, the flow rate of 2 μL s^–1^ was selected for further assays, providing fast analysis without compromising detection performance.

#### Effect of Sample Volume

**Figure 1:**
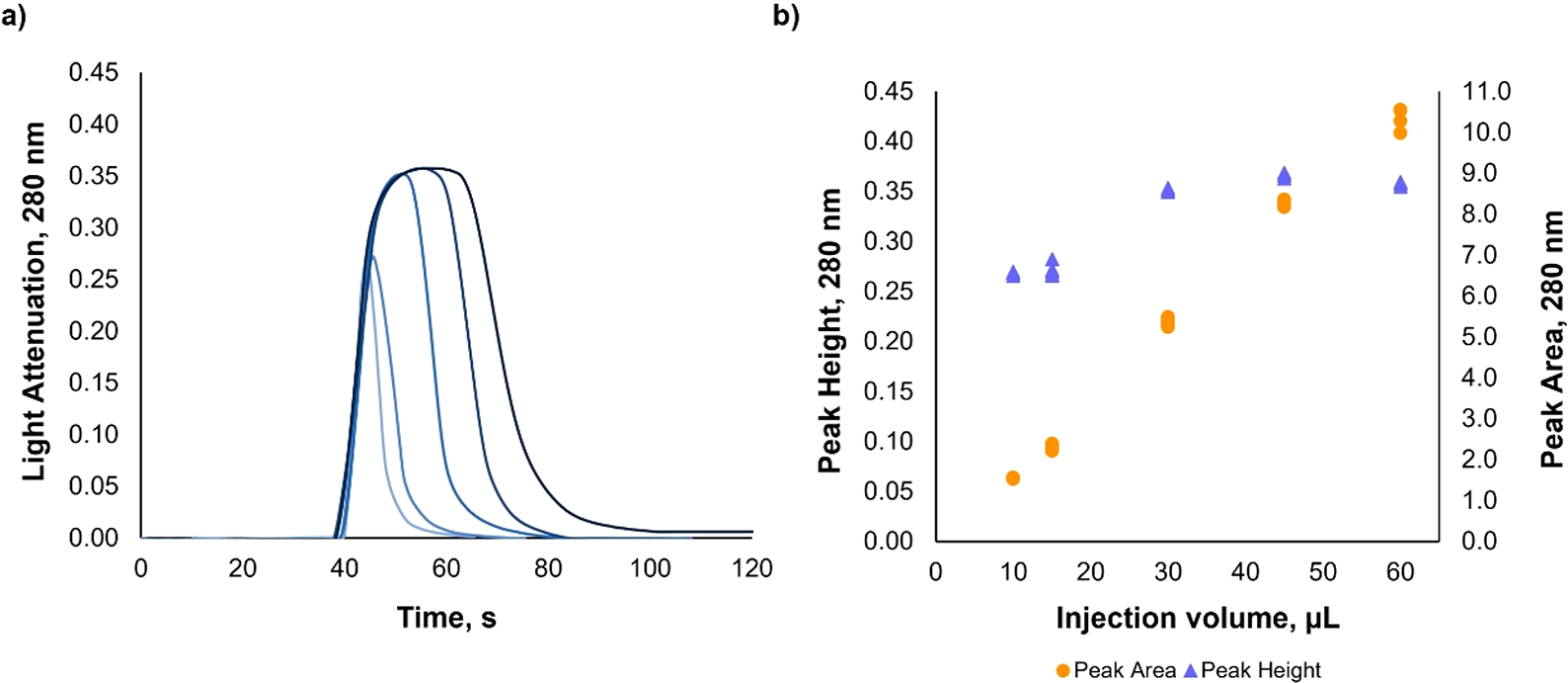
Effect of sample volume on analytical readout. (a) Signal profile (light attenuation 280 nm vs time, color darkening representing increasing volumes) and (b) maximum peak height (blue triangle) and peak area (orange circle) for different volumes of a suspension containing polystyrene NPs A (188 ± 4 nm) at 2 × 10^10^ particles mL^–1^ (*n* = 5, RSD values < 2% for peak height and <3% for peak area).

Different volumes (10–60 μL) of polystyrene NPs A were sent to the detection unit to select the best conditions for quantitative measurements. A marked increase (≈25%) in peak height (light attenuation vs. time) was observed with increasing sample volume up to 30 μL (Figure 1). For volumes > 30 μL, peak height was not markedly enhanced. In opposition, a linear increase of the peak area for increasing volumes was observed, following the linear relation *peak area* = 0.181 (±0.003) × *sample volume* – 0.24 (±0.11), (*R*^2^ = 0.9941, *sample volume* expressed in μL), suggesting that NPs did not agglomerate in the carrier plug when passing through the LOV detection unit for the range of tested volumes. A similar effect was described by Ruzicka et al. when small molecules (<3 nm) were analyzed in the LOV flow system:^33^ when no increase in peak height was observed, the central segment of the sample was not diluted in the carrier (Ruzicka’s dispersion coefficient = 1).

Thus, the same principles were verified herein for NPs analyzed in a flow system. Considering the results obtained, 30 μL was selected as the sample volume for further assays, providing a good compromise among sensitivity, sample spending, and time of analysis (120 s per determination).

#### Effect of Nanoparticle Concentration and Size

The flow conditions set above were applied to evaluate analytical readout for increasing NPs concentrations. A linear increase in peak height was observed for increasing concentrations of polystyrene NPs A (light attenuation 280 nm= 1.70 (±0.01) × 10^–11^ [NPs A] – 0.002 (±0.001), *R*^2^ ≥ 0.9996 where [NPs A] is given in number of particles mL^–1^, Table S2). Similarly, a linear increase in peak area was also observed (Table S2), while peak width was constant along all the concentrations tested (Table S3), confirming the reproducible dispersion of NPs using the LOV platform.

Intermediate precision values for peak height (RSD ≤ 3.6%) and peak area (RSD ≤ 11.3%) (*n* = 10) were aligned with the required by bioanalytical method guidelines,^29^ proving the method suitable for quantification purposes.

**Figure 2:**
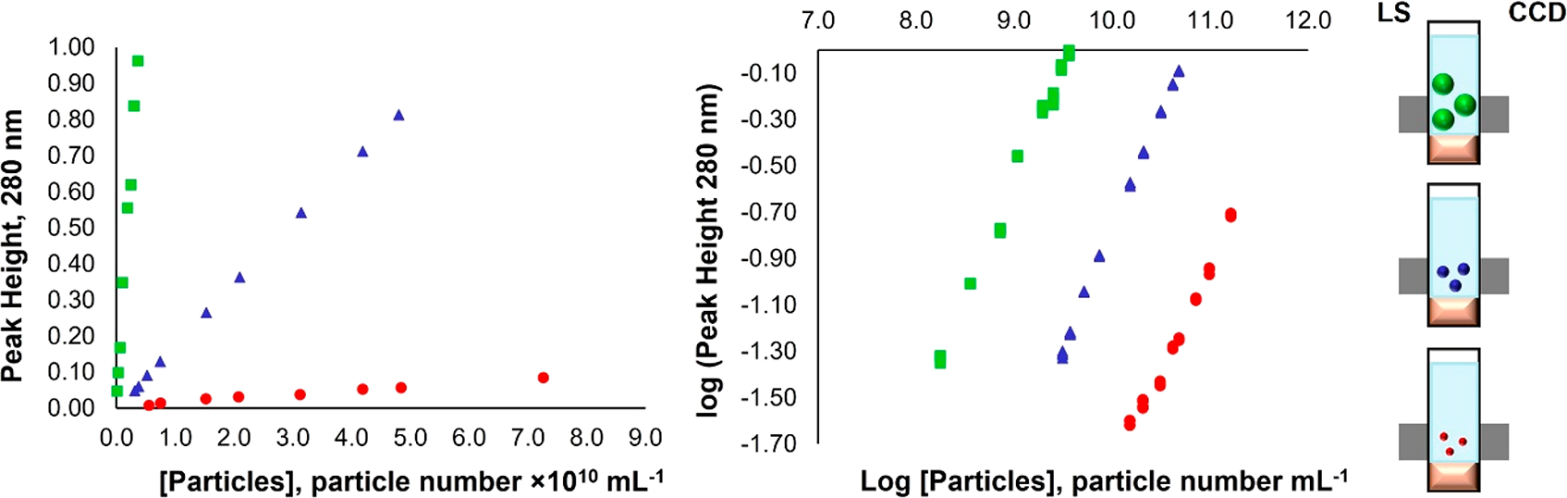
Peak height at 280 nm for different concentrations of polystyrene NPs with a diameter of *ca*. 500 (green square), 200 (blue triangle), and 100 (red circle) nm (*n* = 5) (left panel), and the logarithmic representation of this correlation (middle panel). Schematic representation of the volume occupied by the same number of NPs of different diameters (right panel). LS, light source; CCD, spectrometer.

When the same study was performed with polystyrene NPs with diameters of 102 ± 3 (NPs B) and 502 ± 13 nm (NPs C), a linear increase in peak height and area for increasing concentrations of NPs B and C was also observed, demonstrating the method feasibility to distinguish between NPs concentrations for NPs within 100–500 nm size range (Table S2, Figure 2).

However, the working range and sensitivity for these NPs was different than the obtained for NPs A (Figure 2, Table S2). Indeed, when the NP size was ≈2× smaller to that of NPs A, a decrease of ≈15× in the sensitivity was obtained for peak height and area. The different sensitivity attained for NPs of the same material, density and concentration but with different sizes is a result of the increase in the light scattered when particle size increases.^35,36^ Indeed, the ratio (light attenuation/particle concentration) is directly proportional to the extinction efficiency (*Q*_ext_), and to the square of particle diameter, as described by Mie theory (equation S1) when multiple scattering is not verified (as occurs in the concentration ranges used for NPs A and B).^37^ For NPs B, the squared diameter and the *Q*_ext_ are ≈3× and ≈5×^36^ lower in relation to that of NPs A, thus corresponding to the ≈15× sensitivity decrease observed experimentally.

On the contrary, when NPs size was ≈2.7× higher in relation to that of NPs A (500 nm), an increase of ≈15× in the sensitivity has also been observed. In this case, the sensitivity was 2× higher than the theoretically expected considering the *Q*_ext_ values given by Mie theory and size values (theoretical value NPs C/NPs A ≈ 7.0; experimental value NPs C/NPs A ≈ 15.0). However, it has been described that when NP size increases, deviations from the expected Mie theory *Q*_ext_ values occur.^16,38,39^ Indeed, deviations of ≈2× have been described for NPs with a size higher than that of the incident wavelength. These have been attributed to the increase of particle’s geometrical cross section, that affects *Q*_ext_ values,^38,39^ which could possibly justify the increased sensitivity attained.

Additionally, peak width was constant for all the NPs (A, B, C) tested (differences ≤ 9.6% in peak width for NPs A, B, and C analyzed at the same concentration level), and thus, this parameter was not influenced by NPs size. Hence, the gathered results confirm that transport within the flow conduits was not affected by NP size and concentration, and that the analytical signal (decrease in the light received by the detector due to scattered light) was proportional to NPs concentration for NPs of the same material and size and with monodisperse distributions (i.e., only one size population), being aligned with the NPs light scattering fundamentals described (Supporting Information, Section 2). Indeed, although NPs A, B, and C have the same overall density (1.05 g cm^–3^), when the same number of NPs is sampled from each formulation and dispersed in 1 mL, a different volume will be occupied by the particles because of their differences in size,^7^ resulting in different scattering intensities (Figure 2).

Thus, the present method was responsive to NPs concentration, enabling to distinguish concentrations between batches of particles of the same material and with similar particle sizes. Still, independent particle size measurements must be performed prior to analysis for adequate result interpretation.^9^ Likewise, the analysis of a particle calibrant at each day of analysis (*e.g.*, at the beginning and/or end of the day) for system assessment is recommended, which is a usual practice in flow analysis systems, considering its three principles for reliable, nonequilibrium determinations: (i) repeatable insertion of sample into a flowing stream, (ii) controlled dispersion of sample in the carrier phase, and (iii) reproducible timing of events, including sampling and detection.

### Application to PEG–PLGA Nanoformulations

#### PEG–PLGA Nanoparticles

PEG–PLGA NPs are among the most produced particles for drug-delivery aims, as PEG–PLGA is an FDA approved polymer, with biodegradable, biocompatible and stealth properties presenting a safe and long-circulation profile.^26^ For organic NPs holding a core and surface with different compositions (and densities) as these, the expression of NPs dose as NPs number mL^–1^ in detriment of mass of polymer mL^–1^ is highly recommended for a correct interpretation of cytotoxicity/efficacy results, especially when comparisons with other formulations are envisioned during NPs development stages.^7^

Thus, the proposed method was applied to quantify a PEG–PLGA NPs formulation with 133 ± 2 nm and monodisperse distribution (Figure S4). A linear increase of peak height and area was observed for increasing concentrations (0.139–1.39 × 10^12^ particles mL^–1^) of PEG–PLGA NPs (Figure S5, Table 1).

**Table 1:** Figures of Merit for PEG–PLGA and PEG–PLGA–MTX NPs Concentration Measurements

	PEG–PLGA	PEG–PLGA-MTX
calibration curve		
peak height 280 nm vs. [NPs]^a^	Slope: 3.42 (±0.06) × 10^–13^	Slope: 3.35 (±0.04) × 10^–13^
	Intercept: −0.014 (±0.005)	Intercept: −0.003 (±0.004)
	*R*^2^ > 0.9915	*R*^2^ > 0.9964
peak area 280 nm vs. [NPs]^a^	Slope: 7.0 (±0.2) × 10^–12^	Slope: 7.2 (±0.2) × 10^–12^
	Intercept: −0.5 (±0.2)	Intercept: −0.5 (±0.2)
	*R*^2^ > 0.9726	*R*^2^ > 0.9826
working range^a^	1.39–13.9 × 10^11^	2.01–20.1 × 10^11^
intermediate precision		
peak height	2.0–12%	2.3–8.6%
peak area	6.2–13%	6.0–15%
LOD^a^	0.2 × 10^11^	0.2 × 10^11^
LOQ^a^	0.4 × 10^11^	0.4 × 10^11^

a Results expressed in number of particles mL^–1^.

As observed for polystyrene NPs, differences <6% in peak width were obtained along all the tested concentrations (Figure S5). Intraday RSD values were <6% and <10% for peak height and peak area, respectively. Likewise, interday RSD values were ≤13%.

However, the sensitivity of these determinations was lower (≈ 50× lower slope) in relation to the achieved for polystyrene NPs with a similar size (NPs A, Table S2). This is consistent with light scattering fundamentals, considering the lower refractive index of PEG and PLGA (1.46–1.47)^40,41^ in relation to that of polystyrene (1.59–1.62).^30,42^ Indeed, the intensity of the scattered light will be lower when smaller differences exist between the refractive index of the NPs and that of the medium (≈1.34–1.35).^30^ This is further amplified by the size differences between the PEG–PLGA NPs and the polystyrene NPs A under study (NPs A size is ≈30% higher than that of PEG–PLGA NPs), justifying the different scattering intensities attained.

Therefore, the achieved results highlight the need for correlating the scattering values obtained in the LOV with the concentration values measured by other technique, such as PTA (establishment of LOV/PTA signal correlation), the first time a NPs formulation is analyzed in the LOV system, since each NPs formulation has its own physical/light scattering properties. Such would allow to establish a LOV signal/particle concentration reference for subsequent concentration measurements of NPs of the same size and composition that could be performed solely on LOV afterward, due to method reproducible features and using a particle calibrant for daily system assessment as recommended above.

#### Methotrexate Loaded PEG–PLGA Nanoparticles

**Figure 3:**
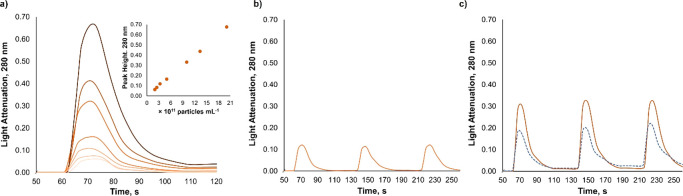
Analytical signal profile (light attenuation 280 nm vs. time) for (a) increasing concentrations of PEG–PLGA–MTX NPs in PBS (correlation of peak height vs. NPs concentration (*n* = 5) in insert), (b) PEG–PLGA–MTX NPs (3rd lower level of a) after incubation in pepsin-free simulated gastric fluid (*n* = 3), and (c) PEG–PLGA–MTX NPs (3rd lower level of a) after incubation in pepsin-containing simulated gastric fluid (orange solid line) vs. signal profile for this fluid in the absence of NPs (blue dashed line) (*n* = 3).

Thereafter, the proposed procedure was applied to a formulation of PEG–PLGA NPs containing an encapsulated drug, methotrexate (MTX), which absorbs light within 200–450 nm (Figure S6). Different concentrations of PEG–PLGA-MTX NPs with 151 ± 3 nm, polydispersity values ≤ 0.07, and with monodisperse distribution (Figure S4) were analyzed rendering a linear correlation between NPs concentration and peak height/area (Figure 3a, Table 1).

However, slight differences in peak height and area were observed between empty and loaded NPs at 280 nm (|*t*_calc_| > 3.70, *t*_tab_ = 2.0, ν > 48, *p* = 0.05, concerning the slopes for analytical signal vs. NPs concentration, Table 1). Increased slopes for peak height vs. NPs concentration were obtained for empty NPs in comparison with loaded NPs at this λ_inc_, despite the smaller size (*ca*. 20 nm) of empty NPs, whereas for peak area higher sensitivity was obtained for loaded NPs (as expected considering the higher NPs size). This trend for peak height at 280 nm was not consistent with the analytical signal obtained at 480 nm (Table S4), a wavelength at which the loaded compound (MTX) does not absorb (Figure S6). At 480 nm, the slope for both peak height/area vs. NPs concentration was significantly higher for loaded NPs in relation to empty ones (|*t*_calc_| > 3.3, *t*_tab_ ≤ 2.1, ν > 26, *p* = 0.05, Table S4). These results suggest that, at 280 nm, part of the light illuminating the particles is absorbed by the loaded compound, with less light available to be scattered by the particles while these pass through the flow cell. Therefore, particle concentration estimations and comparisons between batches that contain loaded compounds must be performed using a λ_inc_ at which the loaded compounds do not absorb light or resorting to peak area values.

Hence, particle concentrations for both empty and loaded NPs could be differentiated by the proposed method. Still, these determinations must be carried out along with particle size measurements for adequate result interpretation. Moreover, NPs light scattering signals must be calibrated in relation to PTA concentration values the first time NPs are analyzed in the LOV system, as discussed above for empty NPs.

### Figures of Merit

The present method was suitable for monitoring NPs concentration in nanoformulations with different compositions (polystyrene, PEG–PLGA and PEG–PLGA–MXT) and sizes (≈100–500 nm).

Working ranges from 10^9^ to 10^11^, 10^9^ to 10^10^, and 10^8^ to 10^9^ particles mL^–1^ were feasible for polystyrene NPs with *ca*. 102, 188, and 502 nm, respectively (Table S2). LOD and LOQ values within 10^7^ and 10^9^ were determined for these NPs (lower values for larger particles, Table S2). Likewise, working ranges for PEG–PLGA and PEG–PLGA–MTX NPs were from 10^11^ to 10^12^ particles mL^–1^, with LOD/LOQ values in the 10^10^ particles mL^–1^ range (Table 1).

The working concentration ranges were higher than the commonly applied for PTA and TRPS analysis (10^7^–10^9^ particles mL^–1^),^5,13^ the most used techniques for NPs counting, thus enabling measurements resorting to less diluted samples (*e.g.*, dilutions of *ca.* 5–50 times for LOV and 16000 times for PTA regarding the analysis of the PEG–PLGA NPs under study). This is advantageous due to the instability commonly caused in the nanoformulations by extensive dilutions (such as those required for PTA).^5^

The analysis of a control sample before and after LOV analysis (by collection of the fluid leaving the flow cell) revealed no significant differences in NPs concentration (|*t*_calc_| = 1.91, *t*_tab_ = 2.20, ν = 11, *p* = 0.05) and size (|*t*_calc_| = 0.56, *t*_tab_ = 2.20, ν = 11, *p* = 0.05) (Figure S7) as confirmed by PTA. This suggests that NPs integrity was unaffected during analysis, and that there was no detectable NP loss in the flow system. This is aligned with LOV system features, namely, the inert and relatively large bore (*ca*. 1.6 mm) conduits when compared to microfluidic systems. Furthermore, analysis was accomplished using flow rates close to 2 μL s^–1^ and without submitting the particles to extensive dilutions in the system (30 μL of sample are recovered in a final volume of 100 μL), thus maintaining NPs properties, in opposition to the commonly verified in column-based procedures (*e.g.*, liquid- or size exclusion chromatography).

Finally, each sample was analyzed within 10 min (for *n* = 5), making the analysis of 6 samples h^–1^ feasible, which is also advantageous considering other NPs concentration measurement strategies (*e.g*., 28–31 min for organic NPs measurements by TRPS).^12^

Besides the above-mentioned advantages, NPs measurements under LOV dismiss experienced operators, optimization of analysis parameters between samples, or laborious calibration procedures (as required for TRPS, for instance).^9^ In fact, quantification by the proposed procedure only requires the characterization of NPs concentration by an independent method the first time they are analyzed at the LOV as a reference, along with knowledge concerning NPs size, providing a more straightforward NP concentration measurement in relation to both PTA and TRPS for routine control operations. Moreover, LOV is a commercially available and easily transportable platform. This, combined with the fact that the proposed method does not require complex post-calculations neither information regarding NPs refractive index, renders the developed method expedite in relation to other currently available procedures (i.e., MALS, DCS) for regular concentration measurements of NPs from distinct materials/composition, expanding the available toolset with a fast, user-friendly, easily implementable, and reproducible procedure for NPs concentration control. In addition, the proposed method is not destructive, allowing NPs reuse for further characterization experiments upon their elution from the LOV, as it does not cause alterations in NPs size (Figure S7).

### Nanoparticle Quantification in Simulated Biological Media

During the development of a new formulation, the evaluation of NPs stability (i.e., intactness) in biological media, such as gastric fluid is required, for instance, when administration by the oral route is aimed. Still, concentration measurements of organic NPs in biological fluids pose some challenges, as counting of protein aggregates as NPs have been reported for TRPS and PTA measurements,^43^ with consequent overestimation of NPs number. Therefore, the feasibility of the developed method to determine PEG–PLGA–MTX NPs concentration upon their exposure to gastric and intestinal simulated fluids was exploited and results compared to those from PTA measurements. Recoveries within 102–115% were determined by direct analysis of PEG–PLGA–MTX NPs after their incubation with simulated gastric fluid (pH 1.2, containing or not pepsin) at 37 °C (Figure 3), suggesting no NPs disruption when these were exposed to this acidic media. No changes in NPs size were detected (relative deviation values < 15%) by PTA, even in pepsin-containing media. Therefore, despite the detection in a wavelength (280 nm) where pepsin may absorb radiation, reliable nanoparticles determination was achieved by subtracting the fluid blank signal at the same wavelength.

Likewise, NPs concentration measurements after NPs exposure to pepsin-free gastric fluid by LOV were consistent (relative deviation values of 11 ± 4%) with the values determined by PTA. Thus, NPs stability upon exposure was confirmed by the developed method and validated by PTA measurements, suggesting the stability of the studied PEG–PLGA–MTX NPs under gastric pH.

Still, PTA measurements failed estimating NPs concentration in pepsin-containing gastric media, providing a marked increase (≈1.5×) in NPs concentration values in relation to the same NPs concentration in protein-free gastric fluid. This increase in NP concentration is likely attributed to PTA bias when measuring NPs dispersed in media containing protein,^9,43^ as strongly suggested considering the results attained with the LOV method (Figure 3c), for which a relative deviation < 10% was obtained in relation to determinations in pepsin-free gastric fluid (Figure 3b).

NPs stability upon incubation in enzyme-free simulated intestinal fluid (pH 6.8) for 4 h at 37 °C resulted in recoveries of 103 ± 1% (Figure S8), suggesting the absence of NPs disruption under these conditions. Similar findings were verified by PTA, with no marked changes in NPs concentration values (|*t*_calc_| = 0.78, *t*_tab_ = 2.4, ν > 7, *p* = 0.05) after 4 h of incubation in this fluid. These results were further supported by the maintenance of NPs size upon incubation (163 ± 2 vs. 169 ± 3 nm).

## Conclusions

In this work, a miniaturized, simple, fast, and nondestructive procedure for NPs quantification based on optical measurements was developed under the SI-LOV platform. The set method allowed reproducible measurements of NPs concentration, for NPs made of polystyrene (100–500 nm), and for PEG–PLGA (≈130 nm) and PEG–PLGA NPs loaded with a therapeutic agent (≈150 nm). Low sample consumption (30 μL) and fast analysis (6 samples h^–1^, *n* = 5) were feasible, with no changes in particle size and concentration being found when eluted fractions were analyzed by PTA.

Additionally, quantification of NPs concentration when these were dispersed in simulated gastric fluid was accomplished, dismissing sample pretreatment upon NPs incubation with this fluid (direct analysis), providing a simple tool to evaluate NPs suitability for gastric passage during formulation development stages.

The simplicity of the proposed procedure makes this method advantageous in relation to other strategies, such as PTA, TRPS, and MALS, for routine quality control operations. This, combined with the portable features of LOV along with its multiple sampling ports (6), renders the method promising for implementation in routine evaluations of NPs concentration at development laboratories. Furthermore, the set method provides a new analytical tool for measuring nanoparticle concentration, affording a means for result validation, as required due to the variability of results reported among techniques.

The gathered results suggest the feasibility of the proposed method for measurements of NPs of different sizes, made of different materials (refractive index ≥ 1.46), only requiring their previous characterization by an alternative method the first time these are analyzed by the LOV method to establish a reference concentration. Additionally, as usual in flow injection systems, daily calibration is recommended.

Future studies on NPs presenting refractive indexes and/or diameters dissimilar from those tested herein can further extend method applicability for NPs characterization at industrial level, for instance, to evaluate the reproducibility of NPs batches, to screen for alterations throughout NPs storage, lyophilization or sterilization, and even to evaluate NPs stability when present in biological fluids (*e.g*., serum, saliva, or urine). Likewise, the insertion of size exclusion materials upstream the LOV optical path is currently under consideration toward extending method applicability to formulations containing nonhomogeneous nanoparticle populations.

## Supporting Information Available

Details on materials, methods and data analysis (section S1); information on light scattering fundamentals (section S2); analytical routine (Table S1) and experimental setup (Figure S1); analytical signal (height, area or width) vs. NPs concentration at 280 nm (Tables S2, S3) and at 480 nm (Table S4); analytical signal for the studied NPs at different wavelengths (Figure S2), for NPs A at different flow rates (Figure S3), and for PEG–PLGA NPs at increasing concentrations (Figure S5); PTA size distributions for (i) empty and MTX-loaded PEG–PLGA NPs (Figure S4), and (ii) for NPs before and after LOV analysis (Figure S7); MTX absorbance spectra (Figure S6); Light attenuation 280 nm vs. time for PEG–PLGA–MTX NPs incubated with intestinal simulated fluid (Figure S8) (PDF)
