# Supplementary material for: Lab-on-Valve Automated and Miniaturized Assessment of Nanoparticle Concentration Based on Light-Scattering
Source: Anal Chem. 2023 Feb 21;95(10):4619–26. doi: 10.1021/acs.analchem.2c04631 (PMC10018450; doi:10.1021/acs.analchem.2c04631)
Supplement: Supplementary file 1 — ac2c04631_si_001.pdf [file ac2c04631_si_001.pdf]

Supporting Information

Lab-on-Valve Automated and Miniaturized Assessment of Nanoparticle Concentration Based on Light-Scattering

Sara S. Marques[a,*], Inês I. Ramos[a], Carla Silva[b], Luisa Barreiros[a,c], Maria R. Domingues[d,e], Marcela A. Segundo[a,*]

[a]*LAQV, REQUIMTE, Department of Chemical Sciences, Faculty of Pharmacy, University of Porto, R. Jorge Viterbo Ferreira 228, 4050-313 Porto, Portugal.*

[b]*Centre of Biological Engineering (CEB), University of Minho, 4710-057 Braga, Portugal; LABBELS - Associate Laboratory, 4710-057 Braga, Guimarães, Portugal.*

[c]*School of Health, Polytechnic Institute of Porto, R. Dr. António Bernardino de Almeida 400, 4200-072 Porto, Portugal.*

[d]*CESAM-Centre for Environmental and Marine Studies, Department of Chemistry, Santiago University Campus, University of Aveiro, 3810-193 Aveiro, Portugal.*

[e]*Mass Spectrometry Centre, LAQV REQUIMTE, Department of Chemistry, Santiago University Campus, University of Aveiro, 3810-193 Aveiro, Portugal.*

[*]Corresponding authors: scmarques@ff.up.pt; msegundo@ff.up.pt

Abstract: In this supporting information file, additional information on materials, methods and data analysis is provided (section S1), along with information on nanoparticles light scattering fundamentals (section S2). Moreover, elements considered important to complement the information provided in the main file are supplied such as the LOV analytical routine (Table S1), the experimental setup (Figure S1), and additional results, as detailed on the Table of contents (page S-2).

Abbreviation list: λinc, incident wavelength; Q_{ext} , extinction efficiency; µL, microliter; CCD, charge-coupled device; DLS, dynamic light scattering; LOD, limit of detection; LOQ, limit of quantification; LOV, lab-on-valve; MTX, methotrexate; NPs, nanoparticles; NPS A, standard polystyrene NPs with 188 ± 4 nm; NPS B, standard polystyrene NPs with 102 ± 3 nm; NPS C, standard polystyrene NPs with 502 ± 13 nm; PBS, phosphate-buffered saline solution; PEG-PLGA, poly(ethylene) glycol methyl ether-block-poly-D,L-lactide-co-glycolide; PEG-PLGA-MTX, PEG-PLGA NPs loaded with methotrexate; PTA, particle tracking analysis; PVA, polyvinyl alcohol; SI, sequential injection; SI-LOV, sequential injection lab-on-valve; UV-vis, ultraviolet-visible spectrophotometry.

Table of contents

S1. Supporting materials and methods S3

S2. Supporting results and discussion S7

S3. Supporting Tables S9

Table S1. SI-LOV analytical routine for nanoparticle (NP) quantification S9

Table S2. Figures of merit for determinations of polystyrene NPs concentration S10

Table S3. Peak height, area and width according to NPs concentration at 280 nm S11

Table S4. Peak height/area *vs.* NPs concentration for PEG-PLGA and PEG-PLGA-MTX at 480 nm S12

S4. Supporting Figures S13

Figure S1. Configuration of the SI-LOV system for the quantification of NPs S13

Figure S2. Light attenuation *vs*. time for NPs A, B, C and for PEG-PLGA NPs at different wavelengths S14

Figure S3. Light attenuation $_{280\ nm}$ *vs.* time for NPs A sent to the LOV detection unit at different flow rates S15

Figure S4. PTA number-based size distributions for PEG-PLGA and PEG-PLGA-MTX NPs S16

Figure S5. Light attenuation $_{280\ nm}$ *vs.* time for increasing concentrations of PEG-PLGA NPs S17

Figure S6. Absorbance spectra for MTX S18

Figure S7. PTA size distributions for MTX-PEG-PLGA NPs before and after analysis in the LOV S19

Figure S8. Light attenuation $_{280\ nm}$ *vs.* time for PEG-PLGA-MTX NPs incubated with intestinal simulated fluid S20

S5. Supporting References S21

S1. Supporting materials and methods

Reagents and solutions

Poly(ethylene) glycol methyl ether-block-poly-D,L-lactide-co-glycolide (PEG-PLGA), polyvinyl alcohol (PVA) (MW = 13,000 - 23,000, 87-89% hydrolysed), and all buffer salts were purchased from Sigma-Aldrich (St. Louis, MO, USA). Methotrexate (MTX) was kindly supplied by Excella (Feucht, Germany). Acetone (analytical grade) was acquired from VWR Chemicals (Radnor, PA, USA). Ultrapure water (resistivity > 18 MΩ cm, Sartorius, Goettingen, Germany) was used to prepare all solutions.

The phosphate-buffered saline solution (PBS) was prepared by dissolving the composing salts in ultrapure water, resulting in the following composition: 140 mM NaCl, 16 mM Na_2HPO_4, 4 mM KCl, 2 mM KH_2PO_4, 16 mM Na_2EDTA, 10 mM LiCl (pH 7.4).

Simulated biological fluids

The simulated gastric fluid (pH 1.2) was prepared according to the monograph "Test solutions" of U.S. Pharmacopeia 38, adjusting the final volume to the amount required for the assay. [1] For that purpose, 20 mg of NaCl and 32 mg of pepsin were dissolved in 10 mL of water containing 70 µL of HCl commercial solution (37% w/w). The same fluid without pepsin was also prepared. The enzyme-free simulated intestinal fluid (pH 6.8) was prepared using a 0.05 M of potassium phosphate monobasic solution, and a 1 M NaOH solution to adjust pH, according with the U.S. Pharmacopeia 38 "Test solutions" monograph.

Preparation and purification of PEG-PLGA NPs

Twenty mg of PEG-PLGA polymer were dissolved in 1.0 mL of acetone and solubilized with aid of an ultrasonic bath (15 min). Two mg of MTX were added to this organic phase (polymer + acetone). This solution was then added dropwise to 20 mL of 1% (w/v) PVA and submitted to probe-sonication for 1 min (70% amplitude). Acetone was evaporated by

magnetic stirring (300 rpm, room temperature) overnight. Empty NPs were prepared likewise, without MTX addition to the organic phase. After preparation and characterization by DLS (ZetaPALS Particle Analyser, Brookhaven Instrument Corps, Santa Barbara, CA), the NPs were washed in ultrapure water by centrifugation (18 000 ×g, 25 min, at 20 ˚C) twice, to eliminate residual elements from the preparation procedure such as acetone, free MTX, and the excess of surfactant (PVA). The NPs pellet resulting from the washing procedure was redispersed in water for a final NPs suspension with a theoretical concentration of 26.7 mg mL^{-1} of PEG-PLGA (considering the mass of polymer used to prepare the batch and the final volume in which the NPs were redispersed). The hydrodynamic diameter and PDI of the final NPs suspension were newly assessed by DLS and compared to the values found before NPs washing, to inspect for changes caused by the washing procedure. Different concentration levels of these nanoparticles were analysed by adequate dilution in PBS buffer.

Details on the SI-LOV apparatus used in this work

The SI-LOV (MicroSIA, FIAlab instruments, Inc., Bellevue, WA, USA) system was composed by a 1.0 mL bidirectional syringe pump, a two-way solenoid valve, a holding coil (i.d. 0.8 mm, 139 cm length), and the LOV device with its micro machined conduits (1.6 mm i.d.), mounted atop a six-port multiposition valve (Figure S1). [2, 3] The ports were assigned as: 1) sample port; 2) waste; and 3) detection unit. Ports 4-5 were not used. The system was controlled by FIAlab software (FIAlab instruments Inc.) and operated at room temperature.

LOV detection unit is comprised within LOV channel 3, and its preceded by a 0.8 mm i.d. polytetrafluoroethylene tubing (6 mm length, Omnifit, Cambridge, UK) inserted in this conduit upstream the optical path, to ensure a uniform distribution of NPs throughout the optical path [4]. The detection unit (1.6 mm optical path, 1.2 mm height, 2.4 µL volume) holds

a flow-through cell configuration, being interfaced by paired emitter-receptor optical fibers (600 µm) aligned perpendicularly to the flow stream. While passing the flow cell, the NPs were illuminated by the emitter optical fibre, connected to a deuterium-halogen light source (model DH-2000, Top Sensor Systems, Eerbeek, The Netherlands), while the radiation passing through the flow cell was collected by the receptor fibre, placed in the opposite front, connected to a USB4000-UV-vis CCD spectrometer (Ocean Optics, El Dorado Hills, CA, USA) for signal monitoring. The CCD spectrometer was operated with a sample rate of 2 Hz and an integration time of 11 ms, while the parameters "detectors to average" and "scans to average" were defined as 5 and 3, respectively. Light attenuation monitoring was performed at 280, 302, 320 and 480 nm. From these, 280 nm was defined as the major wavelength for data analysis. Concurrently, 480 nm was defined as the reference wavelength as this afforded i) no absorbance by the MTX molecule and ii) $\frac{2\pi r n}{\lambda} < 2.0$ (r, NPs radius; n, media refractive index; λ, wavelength), which imparts reliable predictions by Mie theory for NPs up to 230 nm. A peek plug (0.13 mm i.d., *ca.* 2 mm length) was placed immediately after the flow cell. This configuration is also suited for bead injection analysis and no blocking issues were observed.

Nanoparticle quantification in simulated biological media

PEG-PLGA-MTX NPs (at 6.7 $\times 10^{11}$ particles mL^{-1}) were incubated at 37 ˚C, 300 rpm (ThermoMixer® C with SmartBlock™ 2.0 mL, Eppendorf, Germany) for 2 h with simulated gastric fluid (containing or not pepsin), and for 4 h with the simulated intestinal fluid. After the incubation period, the NPs were directly analysed (no sample-treatment) regarding their concentration by the proposed SI-LOV procedure and by PTA, and regarding their size by DLS and PTA. To correct for potential matrix effects, blank solutions (fluids not spiked with

NPs) were also analysed. Likewise, control experiments with NPs incubated in PBS at the same conditions (time, temperature and agitation) were also performed.

Data analysis

A 7-points moving average filter was applied to the acquired data (light attenuation values *vs.* time). From this distribution, maximum light attenuation values (peak height) were determined. Likewise, peak area was calculated as the area under the curve by integrating the light attenuation signal as a function of the time. Peak width was estimated at half height. Linearity was evaluated by performing calibration curves for each formulation with at least 5 concentration levels (with 5 replicate measurements for each concentration).

The limits of detection (LOD) and quantification (LOQ) were calculated for each type of nanoformulation using the signal-to-noise ratio ($n = 10$), corresponding to the concentrations that resulted in a signal-to-noise of 3:1 and 10:1, respectively.[5] Repeatability and interday precision were evaluated for peak height and area for 3 concentration levels (≥ 5 replicates each). Accuracy (%) was expressed as the percent ratio of the measured NPs concentration and the nominal value determined by PTA. Additionally, method comparison was established using PEG-PLGA-MTX NPs processed both by LOV and PTA.

NPs size and concentration were presented as the mean ± standard deviation. Paired t-test was performed whenever needed for mean comparison. The t-test values ($|t_{calc}|$) were calculated and compared to $t_{tabulated}$ values (t_{tab}) ($p = 0.05$) resorting to Excel software (Microsoft, Redmond, WA, USA).

For assays in complex media (simulated gastric fluid with pepsin), the peak height and the peak area values for the blank (fluid not spiked with NPs) were determined and subtracted to the signals obtained for the analysis of NPs incubated in the complex simulated media.

S2. Supporting results and discussion

Nanoparticle quantification based on light scattering under dynamic flow injection – fundamentals

Fundamentals on NPs light scattering

When NPs are illuminated by a light beam, part of the incident light is scattered in all directions. [6, 7] The total light scattered by a set of particles corresponds to the sum of the light scattered by each particle present in the optical path when adequate dilution conditions, that promote enough distance among particles, are applied (negligible multiple scattering). [6, 8-10] Additionally, the light scattered by a particle is a function of i) the differences in the refractive indexes of the particle and the media, ii) the wavelength of illumination (whose magnitude is inversely proportional to scattering intensity [7]), and iii) particle properties, such as size, concentration, and shape (*i.e.*, sphericity). Considering nanoparticles size, different scattering theories may apply: *Rayleigh scattering,* for particles with a diameter at least 10 times lower than the incident wavelength (λ_{inc}); *Rayleigh approximation*, for larger particles than those encompassed by the last theory but still significantly smaller in relation to λ_{inc} (valid when $\frac{2\pi r n}{\lambda inc} < 0.8$, r representing particle radius*;* n, medium refractive index*); Mie theory*, for larger particles that do not largely surpass λ_{inc}; and *Fraunhofer diffraction*, for particles ≥ 40 times [10] larger than λ_{inc} [7, 9, 11] Considering that in this work incident wavelengths from 280 – 480 nm and NPs with 100 – 500 nm of diameter were targeted, along with the specifications of the scattering theories detailed above, scattering events were putatively considered to obey to Mie theory. Under Mie theory, the total light scattered is described by Equation S1,

$$\textbf{Light scattering } (\lambda_{inc}) = \frac{[NPs]\pi D^2 Q_{ext} L}{4 ln_{10}} \qquad \textbf{Equation S1}\ [12]$$

where *[NPs]* corresponds to NPs concentration, *D* to NPs size, Q_{ext} to NPs extinction efficiency, and *L* to optical path length. Therefore, increased light scattering will be verified for NPs at higher concentration levels and/or with larger sizes.

Nevertheless, after analysis of experimental results showing that the light attenuation *vs.* $1/\lambda^4$ provided a linear correlation for different nanoparticles ($R > 0.994$) and considering previous work from Sitar *et al* [13], where the hydrodynamic radius was apparently lower when nanoparticles are submitted to translational motion promoted by the carrier fluid flow rate, application of Rayleigh approximation is feasible to the tested nanoparticles < 200 nm.

S3. Supporting Tables

Table S1. SI-LOV analytical routine for nanoparticle (NP) quantification.

Step	LOV valve position	Flow rate (μL s^{-1})	Volume (μL)	SV position[a]	Description
**1**	-	200	900	In	Aspirate carrier into syringe pump
**2**	3	1	20	Out	Start signal acquisition and perform reference scan
**3**[b]	1	3	10 - 60	Out	Aspirate NPs suspension
**4**[b]	3	2	80 - 130	Out	Send holding coil content to the flow cell
**5**	2	100	530	Out	Stop signal acquisition, wash central channel

[a] "Valve In" refers to syringe pump (SP) connected to the carrier reservoir; "Valve out" refers to SP connected to LOV central channel; [b] steps 3-4 performed five times loop-wise before washing the central channel (step 5).

Table S2. Figures of merit for determinations of polystyrene NPs concentration.

	**A**	**B**	**C**
**Size, nm**	188 ± 4	102 ± 3	502 ± 13
**Calibration Curve**			
Peak height $_{280nm}$ *vs.* [NPs][a]	Slope: 1.70 (± 0.01) $\times10^{-11}$<br>Intercept: -0.002 (± 0.001)<br>$R^2 > 0.9996$	Slope: 0.114 (± 0.001) $\times10^{-11}$<br>Intercept: -0.003 (± 0.001)<br>$R^2 > 0.9969$	Slope: 26.6 (± 0.4) $\times10^{-11}$<br>Intercept: 0.009 (± 0.008)<br>$R^2 > 0.9927$
Peak area $_{280nm}$ *vs.* [NPs][a]	Slope: 2.76 (± 0.02) $\times10^{-10}$<br>Intercept: -0.15 (± 0.04)<br>$R^2 > 0.9986$	Slope: 0.177 (± 0.002) $\times10^{-10}$<br>Intercept: -0.03 (± 0.01)<br>$R^2 > 0.9955$	Slope: 42.0 (± 0.1) $\times10^{-10}$<br>Intercept: -0.28 (± 0.16)<br>$R^2 > 0.9911$
Working range [a]	0.31 – 4.8 $\times10^{10}$	0.56 – 16$\times10^{10}$	0.018 – 0.37 $\times10^{10}$
LOD [a]	3×10^{8}	1×10^{9}	2×10^{7}
LOQ [a]	7×10^{8}	2×10^{9}	4×10^{7}

[a] NPs concentration given in number of particles mL^{-1}.

Table S3. Peak height, area and width according to NPs[a] concentration at 280 nm.

[Particles][b]	Peak height	Peak area	Peak width[c]
**0.312 ×10^{10}**	0.049 ± 0.001	0.67 ± 0.03	13.6 ± 0.3
**0.374 ×10^{10}**	0.060 ± 0.001	0.86 ± 0.02	13.8 ± 0.4
**0.519 ×10^{10}**	0.091 ± 0.001	1.29 ± 0.01	15.3 ± 0.1
**0.742×10^{10}**	0.130 ± 0.001	1.95 ± 0.06	14.6 ± 0.7
**1.52 ×10^{10}**	0.265 ± 0.003	4.04 ± 0.09	13.7 ± 0.2
**2.09 ×10^{10}**	0.363 ± 0.004	5.6 ± 0.1	13.9 ± 0.6
**3.14 ×10^{10}**	0.542 ± 0.005	8.6 ± 0.2	14.4 ± 0.3
**4.19 ×10^{10}**	0.712 ± 0.006	11.4 ± 0.4	14.2 ± 0.4
**4.80×10^{10}**	0.813 ± 0.004	13.1 ± 0.3	14.2 ± 0.6

[a] 30 µL of polystyrene NPs A were sent to LOV detection unit at 2 µL s^{-1}.

[b] Particle number mL^{-1}.

[c] Peak width at half height.

Table S4. Peak height/area *vs.* NPs concentration for PEG-PLGA and PEG-PLGA-MTX at 480 nm.

	**PEG-PLGA**	**PEG-PLGA-MTX**
**Calibration curve**		
Peak height $_{480\ nm}$ *vs.* [NPs] [a]	Slope: 5.21 (± 0.08) ×10^{-14}<br>Intercept: –0.001 (± 0.013)<br>R^2 > 0.9924	Slope: 5.79 (± 0.06) ×10^{-14}<br>Intercept: – 0.0003 (± 0.0007)<br>R^2 > 0.9965
Peak area $_{480\ nm}$ *vs.* [NPs] [a]	Slope: 1.11 (± 0.03) ×10^{-12}<br>Intercept: –0.06 (± 0.02)<br>R^2 > 0.9863	Slope: 1.31 (± 0.03) ×10^{-12}<br>Intercept: – 0.10 (± 0.04)<br>R^2 > 0.9865

[a] Number of particles mL^{-1}, as determined by particle tracking analysis.

S4. Supporting Figures

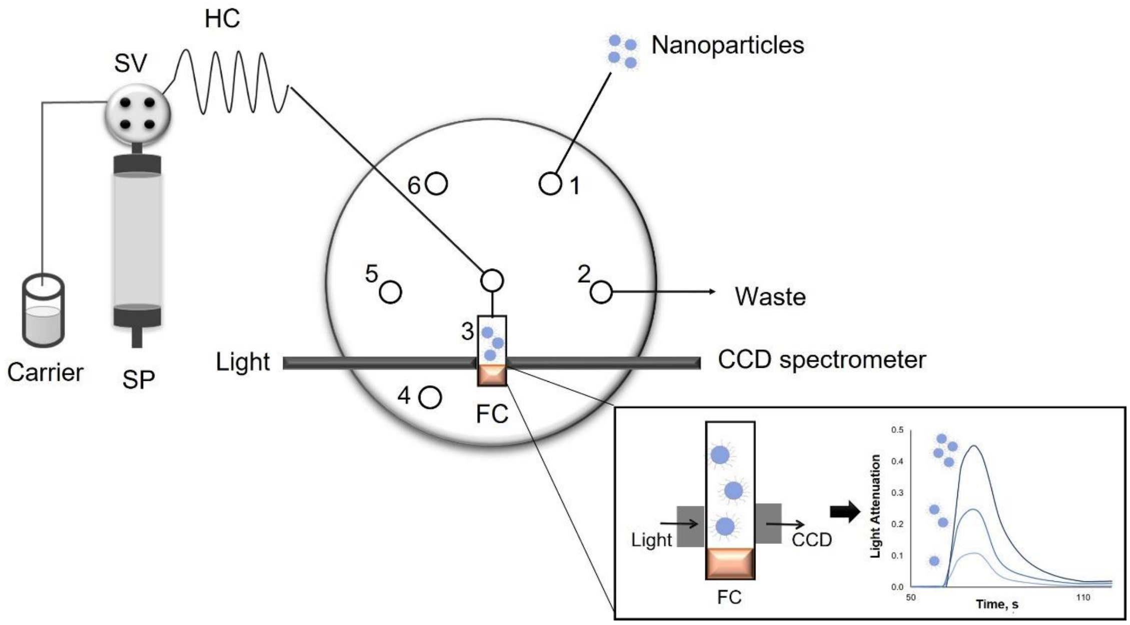

Figure S1. Configuration of the SI-LOV system for the quantification of NPs. NPs suspensions were aspirated from port #1 (3 µL s^{-1}) and sent to the LOV detection unit (flow cell) at 2 µL s^{-1} (port #3) with monitoring of the light attenuation at 280, 302, 320 and 480 nm. 1-6, LOV ports; SP, syringe pump; SV, solenoid valve; HC, holding coil; FC, flow cell; CCD, spectrometer. Phosphate-buffered saline buffer (pH 7.4) was used as carrier.

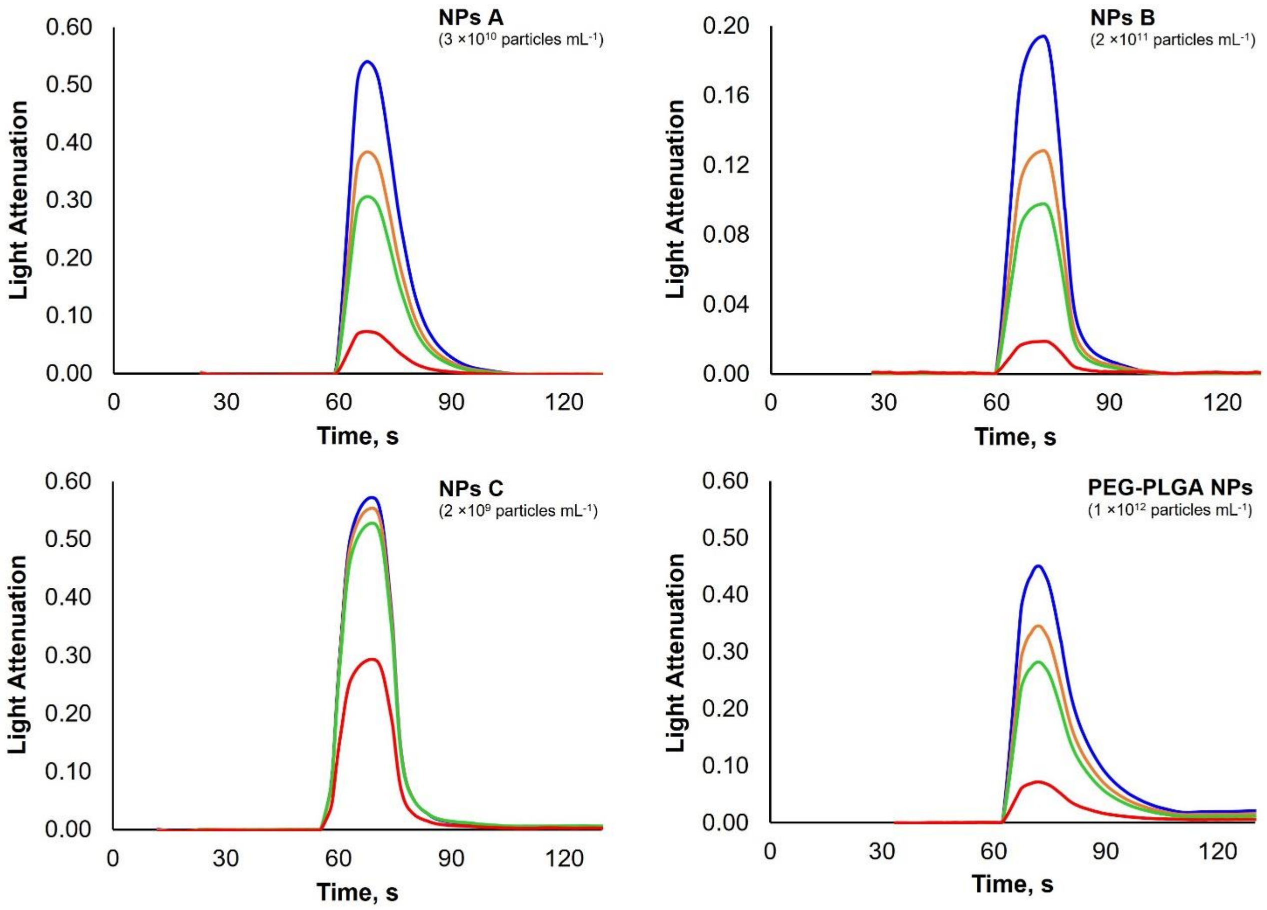

Figure S2. Analytical response (light attenuation *vs.* time) for the polystyrene NPs (NPs A-C) and PEG-PLGA NPs under study at 280 nm (blue line), 302 nm (orange line), 320 nm (green line) and 480 nm (red line).

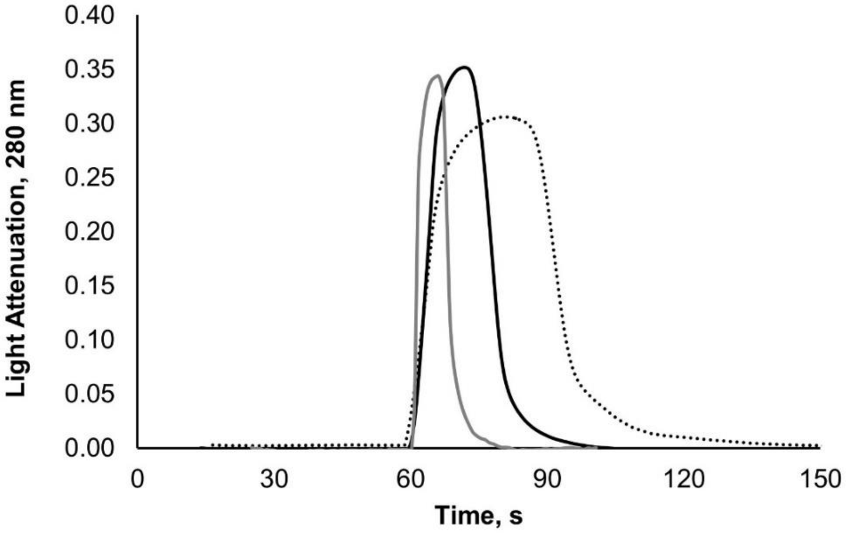

Figure S3. Signal profile (light attenuation $_{280\ nm}$ *vs.* time) obtained when 30 μL of a suspension of polystyrene NPs A (188 ± 4 nm) at 2.17 ×10^{10} particles mL^{-1} was sent to the LOV detection unit at 1.0 (black dotted line), 2.0 (black solid line) and 4.0 μL s^{-1} (grey solid line).

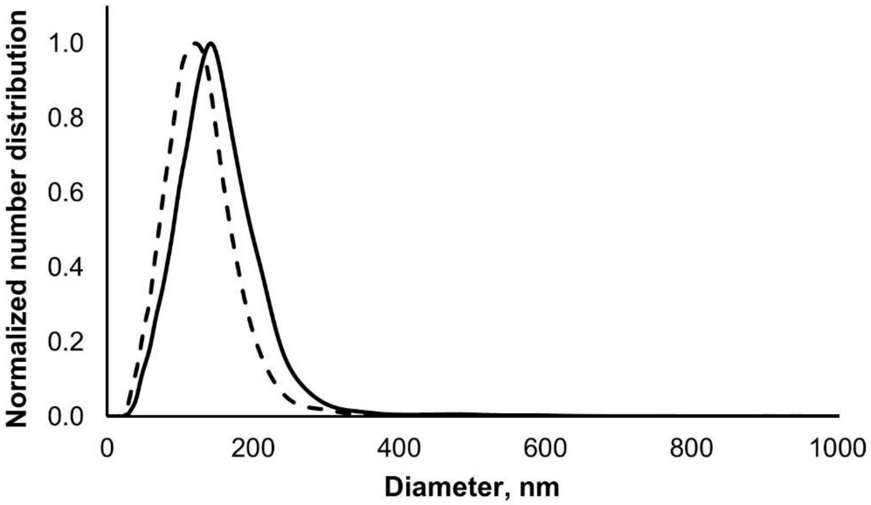

Figure S4. Normalized number-based size distributions obtained by PTA for PEG-PLGA (dashed line) and PEG-PLGA-MTX (solid line) NPs.

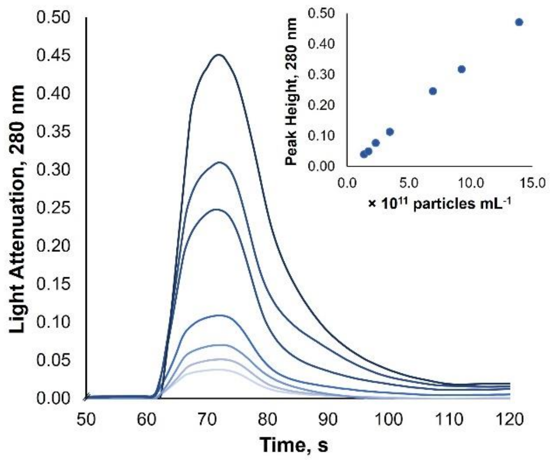

Figure S5. Signal profile (light attenuation $_{280\ nm}$ *vs.* time) for increasing concentrations of PEG-PLGA NPs (n = 5). The corresponding correlation of peak height *vs.* NPs concentration is depicted as insert.

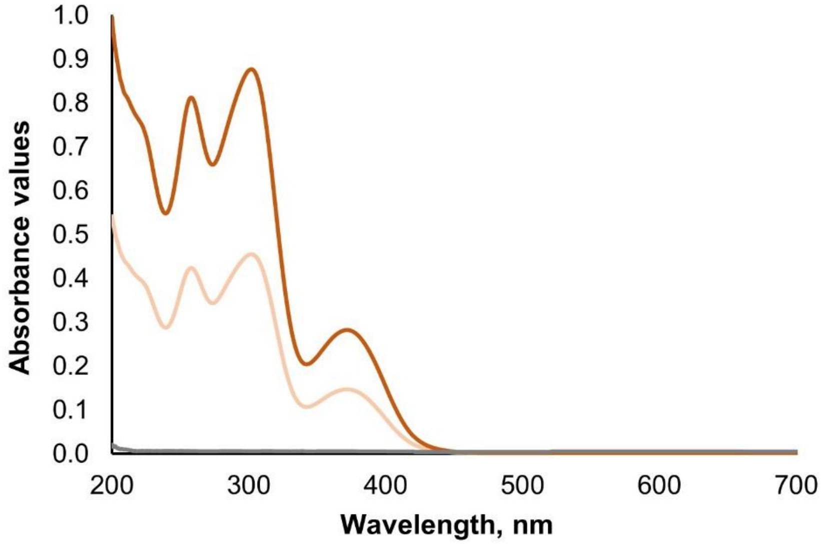

Figure S6 Absorbance spectra for MTX solutions at 10 (light orange) and 20 (dark orange) µg mL^{-1}. Signal for blank solutions (grey) are shown for comparison purpose.

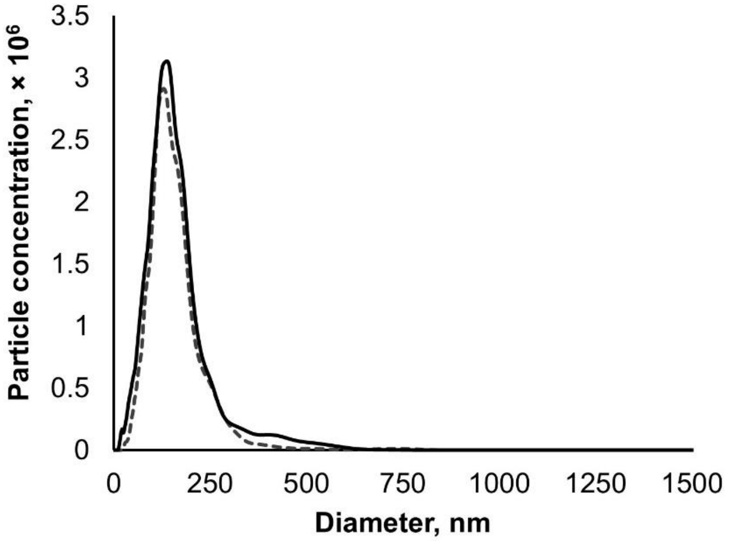

Figure S7. Mean number-based size distributions obtained by PTA for MTX-PEG-PLGA NPs before (dashed line) and after (solid line) analysis in the LOV.

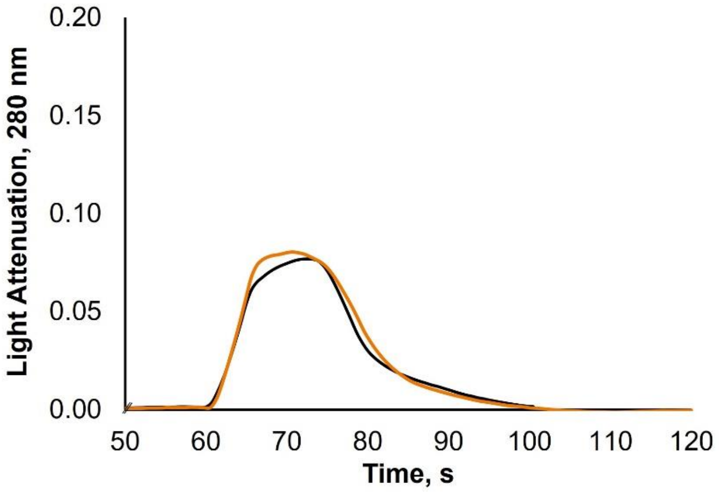

Figure S8. Signal profile (light attenuation $_{280\ nm}$ *vs.* time) for PEG-PLGA-MTX NPs after incubation for 4 h at 37 ˚C with intestinal simulated fluid (pH 6.8, orange line). The signal for the same concentration of NPs before incubation is depicted (black line) for comparison.

S5. Supporting References

(1) U.S. Pharmacopeial Convention. Reagents, indicators, and solutions. In *United States Pharmacopeia and National Formulary (USP 38 - NF 33)*, Rockville, MD, USA, 2015; pp 1809-1902.

(2) Ramos, I. I.; Carl, P.; Schneider, R. J.; Segundo, M. A. Automated lab-on-valve sequential injection ELISA for determination of carbamazepine. *Anal. Chim. Acta* **2019**, *1076*, 91-99, DOI: 10.1016/j.aca.2019.05.017.

(3) Ramos, I. I.; Marques, S. S.; Magalhaes, L. M.; Barreiros, L.; Reis, S.; Lima, J. L. F. C.; Segundo, M. A. Assessment of immunoglobulin capture in immobilized protein A through automatic bead injection. *Talanta* **2019**, *204*, 542-547, DOI: 10.1016/j.talanta.2019.06.023.

(4) Ruzicka, J.; Carroll, A. D.; Lahdesmaki, I. Immobilization of proteins on agarose beads, monitored in real time by bead injection spectroscopy. *Analyst* **2006**, *131* (7), 799-808, DOI: 10.1039/b603768b.

(5) *EMA-ICH. ICH guideline M10 on bioanalytical method validation - Step 2b. EMA/CHMP/ICH/172948/2019. 2019.* https://www.ema.europa.eu/en/documents/scientific-guideline/draft-ich-guideline-m10-bioanalytical-method-validation-step-2b_en.pdf (accessed 2022-11-15).

(6) Bohren, C. F.; Huffman, D. R. Part 1 - Basic theory: Introduction. In *Absorption and Scattering of Light by Small Particles*, Bohren, C. F., Huffman, D. R. Eds.; WILEY- VCH Germany, 1998; pp 1-11.

(7) Light Scattering. In *Particle Characterization: Light Scattering Methods*, Scarlett, B. Ed.; Springer Netherlands, 2002; pp 56-110.

(8) Austin, J.; Minelli, C.; Hamilton, D.; Wywijas, M.; Jones, H. J. Nanoparticle number concentration measurements by multi-angle dynamic light scattering. *J. Nanopart. Res.* **2020**, *22* (5), 108, DOI: 10.1007/s11051-020-04840-8.

(9) Niskanen, I.; Forsberg, V.; Zakrisson, D.; Reza, S.; Hummelgård, M.; Andres, B.; Fedorov, I.; Suopajärvi, T.; Liimatainen, H.; Thungström, G. Determination of nanoparticle size using Rayleigh approximation and Mie theory. *Chem. Eng. Sci.* **2019**, *201*, 222-229, DOI: 10.1016/j.ces.2019.02.020.

(10) Pei, Y. W.; Hinchliffe, B. A.; Minelli, C. Measurement of the size distribution of multimodal colloidal systems by laser diffraction. *ACS Omega* **2021**, *6* (22), 14049-14058, DOI: 10.1021/acsomega.1c00411.

(11) Lockwood, D. J. Rayleigh and Mie Scattering. In *Encyclopedia of Color Science and Technology*, Luo, M. R. Ed.; Springer New York, 2016; pp 1097-1107.

(12) Shard, A. G.; Wright, L.; Minelli, C. Robust and accurate measurements of gold nanoparticle concentrations using UV-visible spectrophotometry. *Biointerphases* **2018**, *13* (6), 5, 061002, DOI: 10.1116/1.5054780.

(13) Sitar, S.; Vezocnik, V.; Macek, P.; Kogej, K.; Pahovnik, D.; Zagar, E. Pitfalls in Size Characterization of Soft Particles by Dynamic Light Scattering Online Coupled to Asymmetrical Flow Field-Flow Fractionation. *Anal. Chem.* **2017**, *89* (21), 11744-11752, DOI: 10.1021/acs.analchem.7b03251.
